# Development of a nonlinear hierarchical model to describe the disposition of deuterium in mother–infant pairs to assess exclusive breastfeeding practice

**DOI:** 10.1007/s10928-018-9613-x

**Published:** 2018-11-14

**Authors:** Zheng Liu, Aly Diana, Christine Slater, Thomas Preston, Rosalind S. Gibson, Lisa Houghton, Stephen B. Duffull

**Affiliations:** 1 School of Pharmacy, University of Otago, Dunedin, New Zealand; 2 School of Medicine and Public Health, Hunter Medical Research Institute, University of Newcastle, Kookaburra Circuit, Newcastle, NSW 2305 Australia; 3 Department of Human Nutrition, University of Otago, Dunedin, New Zealand; 4 Division of Medical Nutrition, Faculty of Medicine, Universitas Padjadjaran, Bandung, Indonesia; 5 Independent Consultant, London, UK; 6 Scottish Universities Environmental Research Centre, University of Glasgow, Glasgow, UK

**Keywords:** Human milk, Breastfeeding, Deuterium-oxide turnover method, Pharmacokinetics, Bayesian, MCMC, Stan

## Abstract

The World Health Organization recommends exclusive breastfeeding (EBF) for the first 6 months after birth. The deuterium oxide dose-to-the-mother (DTM) technique is used to distinguish EBF based on a cut-off (< 25 g/day) of water intake from sources other than breastmilk. This value is based on a theoretical threshold and has not been verified in field studies. The aim of this study was to estimate the water intake cut-off value that can be used to define EBF practice. One hundred and twenty-one healthy infants, aged 2.5–5.5 months who were deemed to be EBF were recruited. After administration of deuterium to the mothers, saliva was sampled from mother and infant pairs over a 14-day period. Validation of infant feeding practices was conducted via home observation over six non-consecutive days with caregiver recall. A fully Bayesian framework using a gradient-based Markov chain Monte Carlo approach implemented in Stan was used to estimate the cut-off of non-milk water intake of EBF infants. From the original data set, 113 infants were determined to be EBF and provided 1500 paired mother–infant observations. The deuterium saliva concentrations were best described by two linked 1-compartment models (mother and infant), with body weight as a covariate on the mother’s volume of distribution and infant’s body weight on infant’s water clearance rate. The cut-off value was based on the 90th percentile of the posterior distribution of non-milk water intake and was 86.6 g/day. This cut-off value can be used in future field studies in other geographic regions to determine exclusivity of breast feeding practices in order to determine their potential public health needs.

**Electronic supplementary material:**

The online version of this article (10.1007/s10928-018-9613-x) contains supplementary material, which is available to authorized users.

## Introduction

Optimal breastfeeding practices during early infancy reduce morbidity and mortality, and improve infant growth, health and development [1]. Exclusive breastfeeding (EBF) up to 6 months of age is one of the optimal breastfeeding practices recommended by the World Health Organization. EBF is defined as the practice of giving an infant only breastmilk (no other food or water). After 6 months of age the addition of appropriate complementary foods to ongoing breastfeeding is recommended for up to 2 years or beyond [2]. However, despite the well-established benefits of breastfeeding, global rates remain below international targets [3]. Efforts to increase the prevalence of EBF have yielded varying results due, at least in part, to the various methods used to evaluate exclusivity. Estimates of EBF are often based on caregiver recall over a single period 24-h period, or in some cases maternal recall for the whole period of breastfeeding since birth. The validity of these recall methods has been questioned with concerns about EBF rates being overestimated in a population due to self-reporting bias [4, 5], especially in the face of programs delivering intensive behaviour change communication on breastfeeding to mothers [6]. In addition, the 24-h recall method does not capture infants who are given food or drink on days preceding the recall period. Several studies have identified significant levels of misreporting among varying population groups when recall methods are compared to a method based on a dose-to-mother (DTM) deuterium oxide dilution (D_2_O) technique [7–10].

The D_2_O dilution DTM method [11] provides an estimate of infant water intake from breastmilk which then allows any additional water ingestion from non-breastmilk sources to be determined. In this technique, deuterium oxide is given orally to the mother. The D_2_O disperses uniformly throughout the body water pool within a few hours and transferred to her infant through lactation. The disappearance of the isotope from the mother and the infant (sampled from saliva, urine or milk) is monitored over a 14-day period. A standard compartmental model is used to provide a description of the data [11]. Essentially, this method requires back calculation of the likely infant dose from non-breastmilk sources once other sources of input and loss have been accounted. Since it is impossible to accurately account for all non-breastmilk sources using the DTM (or indeed any) method then a cut-off value for the non-breastmilk water source is required. Moore et al. [7] recommended a value of 24.6 g/day of water intake from non-breastmilk sources based on maternal self-reported breastfeeding practices. Individual mother–infant pairs with values lower than this cut-off criterion are determined to be EBF and values above this cut-off as non-EBF. This value has yet to be verified in mother–infant pairs.

This investigation work presented here, is concerned with modelling deuterium exposure in mother–infant pairs and determining the cut-off criterion for determining EBF. The aims of this study were to: (i) develop a population pharmacokinetic model to estimate the apparent volume of non-milk water intake in mother–infant pairs using the D_2_O DTM technique, and (ii) identify a cut-off value of non-milk water intake that is compatible with EBF. In this work a fully Bayesian technique is used in order to enumerate uncertainty in both the model parameters and also the assumptions inherent in the use of the standard compartmental model.

## Methods

This section is divided into five sections: (1) the data used in this study; (2) identification of an appropriate structural model to describe the breastfeeding mass transfer of D_2_O; (3) specification of statistical models for the priors and constants; (4) model development, and (5) determination of an appropriate criterion for non-milk fluid intake to define exclusivity of breastfeeding. Components 1–4 correspond to aim (i) and component 5 to aim (ii).

All modelling was performed within a fully Bayesian framework using Stan (v 2.12.0) via the rstan (Version 2.11.1) interface, compiled on C ++ (GCC 4.6.3) and run with R (version 3.3.1). Details of the Stan method are provided in [12]. In brief, Stan is a Monte Carlo sampling algorithm that uses a No-U-Turn sampler (NUTS) to build a set of likely candidate points that spans the target distribution. The algorithm stops automatically when it starts to double back and retrace its steps. Empirically, the NUTS algorithm performs at least as efficiently, and sometimes more efficiently than a well-tuned standard Hamiltonian Monte Carlo method, without requiring user intervention or costly tuning runs [13]. These methods, similar to Gibbs and Metropolis–Hastings sampling, generate Markov chains that fall within the overall group of Markov chain Monte Carlo (MCMC) techniques which are used to make inferences about the posterior distribution of the parameter(s) in question. Some examples of Bayesian analysis include Lunn et al. [14], Duffull et al. [15], Wendling et al. [16] and Wendling et al. [17] for a review.

A full analysis plan was developed outlining the analysis components below, and summarised briefly in this section.

### Data

A calibration study was conducted in Tanjunsari, Sukasari and Pamulihan, subdistricts of Sumedang in the province of West Java, Indonesia. A total of 121 mother–infant pairs were recruited. Infant inclusion criteria included being a singleton, full term (> 37 weeks gestation) with a birth weight > 2500 g. At the time of enrolment, infants were aged 2.0–5.5 months old, had been identified as EBF and had no identified medical problems, e.g. active tuberculosis, severe anaemia (i.e., haemoglobin [< 90 g/L]) or acute malnutrition (i.e. mid-upper arm circumference less than 115 mm). Written informed consent was obtained from all participating mothers. Ethical approval for the study was granted by University of Otago Human Research Ethics Committee New Zealand (H15/125) and the Health Research Ethics Committee Faculty of Medicine Universitas Padjadjaran, Bandung (081), Indonesia.

Pre-dose baseline saliva samples were obtained from the mother and infant on day 0, after which each mother received orally ~ 30 g deuterium oxide (accurately measured to the nearest 0.01 g) and diluted in ~ 50 g drinking water. Saliva samples (~ 2 mL) were collected by placing small sterile cotton balls in the mouths of the mothers and infants for a few minutes, after which the saliva was expressed from the wet cotton ball using a disposable syringe. Post-dose saliva samples were collected from the mother and infant on days 1, 2, 5, 6, 13 and 14. Duplicate saliva samples (two taken within 1 h; around 30 min) were collected on day 0, either day 5 or 6, and day 14. The duplicate samples were treated as replicate measures and the mean of the two sampling times and saliva concentrations were used in the analysis. The rationale for this approach is presented in Supplement 1. After collection, all saliva samples were centrifuged at 3500 r.p.m. and then stored at − 20 °C prior to analysis. The enrichment of deuterium in saliva samples was measured using Fourier transform infrared spectrometry (FTIR). The limit of quantitation for saliva samples is 20 mg/kg and the limit of detection for saliva samples is 6 mg/kg. The day-to-day (inter-day) and intra-day coefficient of variations (CV%) of measurements are similar to each other with the value of 0.5%.

Mothers were instructed to continue exclusively breastfeeding their infants over the 14-day collection period. In all cases direct observation of feeding practises in the home were performed by trained field assistants who were known and trusted by the mothers and were recruited from the local community. Field assistants were trained to observe the mother–infant pairs in their own homes from 6.00 am until at least 6.00 pm each day to ensure a maximum 12-h observation on three non-consecutive days per week (6 days total) over the 14 day full DTM protocol period. During this 12-h observation period, field assistants recorded, in a diary, the time and duration of each breastfeeding episode and classified each breastfeeding practise as EBF and non-EBF according to the operational definitions [18]. Night-time breastfeeding practises for each 12-h period preceding each observation day were assessed by maternal recall. Unannounced spot-checks were also made by the field assistants on the non-observation days.

### Structural model

**Fig. 1:**
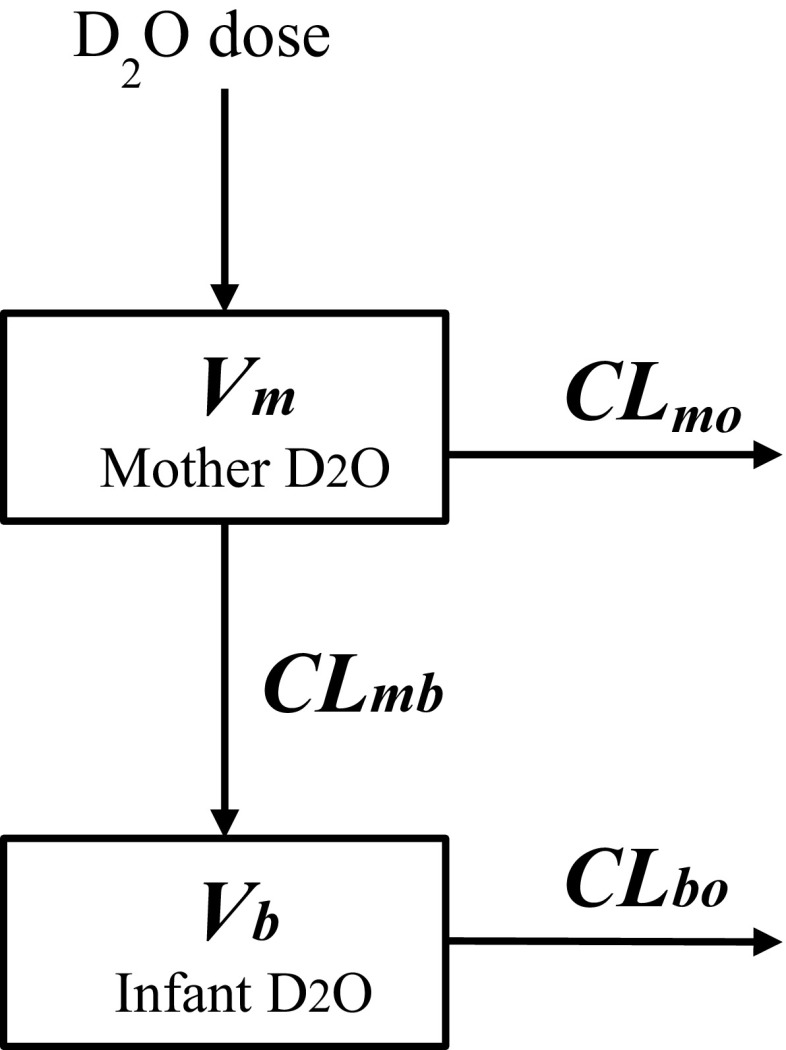
D_2_O disposition model for mother and infant. The term $V$ denotes the D_2_O volume of distribution with subscript *m* and *b* for mother and infant; $CL_{mb}$ is the water clearance from mother to infant; $CL_{bo}$ is the water clearance from infant to out; the term $CL_{mo}$ represents the water clearance from mother to out

The structure model used in this study is a two linked 1-compartment disposition models that represent mother and infant as in Fig. 1. The model is equivalent to those that have been applied previously [11, 25, 27, 28]. Details of the model derivation and assumptions are presented in Supplement 2.

We note that the rate constants $k_{mb}$ and $k_{bo}$ in Eqs. (1) and (2) for D_2_O are identical to H_2_O due to that D_2_O has the same disposition kinetics as H_2_O, and therefore,

1 $$k_{bo} = k_{{bo(H_{2} O)}} = \frac{{CL_{bo} }}{{V_{b} }}$$

2 $$k_{mb} = k_{{mb(H_{2} O)}} = \frac{{CL_{mb} }}{{V_{m} }}$$

and $CL_{bo}$ is the H_2_O clearance rate from infant (units: L/day); $CL_{mb}$ is the H_2_O clearance rate from mother to infant (units: L/day); $V_{b}$ is the D_2_O volume of distribution in infant compartment (units: L); and $V_{m}$ is the D_2_O volume of distribution in mother compartment (units: L). Note here a density of dilute heavy water to be the same as water (= 1.0 kg/L) was applied. The term $CL_{mo}$ in Fig. 1 is the difference of the total clearance from the mother ($CL_{mm}$) and the mothers clearance to the infant and is hence given by $CL_{mm} - CL_{mb}$. Of note, $CL_{mm}$ is replaced by $k_{mm} V_{m}$ in the model and $k_{mm}$ is defined next.

The system expressed as rate constants (for simplicity) is given:

3 $$dA_{m} /dt = - (k_{mm} - k_{mb} )A_{m} - k_{mb} A_{m} = - k_{mm} A_{m} \;({\text{at}}\;t = 0,\;A_{m} = dose)$$

4 $$dA_{b} /dt = k_{mb} A_{m} - k_{bo} A_{b} \;({\text{at}}\;t = 0,\;A_{b} = 0)$$

The analytical solutions of Eqs. (3) and (4) are:

5 $$A_{m(t)} = A_{m(0)} e^{{ - k_{mm} t}}$$

6 $$A_{b(t)} = A_{m(0)} \left( {\frac{{k_{mb} }}{{k_{mm} - k_{bo} }}} \right) (e^{{ - k_{bo} t}} - e^{{ - k_{mm} t}} )$$

In this notation, $A_{m(t)}$ is the mass of D_2_O in mother compartment at time t (units: kg); $A_{m(0)}$ is equal to dose (units: kg); $A_{b(t)}$ is the mass of D_2_O in infant compartment at time t (units: kg); $k_{mm}$ is the rate constant, describing D_2_O total elimination from the mother compartment (units: 1/day); $k_{mb}$ is the rate constant describing D_2_O flow from the mother to the infant via lactation route (units: 1/day); $k_{bo}$ is the rate constant describing D_2_O flow out of the infant compartment (units: 1/day). Notice that the notation $k_{mm}$, is the total D_2_O flow rate constant from the mother, including the exit routes by lactation, urine, breath and skin evaporation.

In this study the concentration of D_2_O is measured, not the mass of D_2_O in the body, therefore Eqs. (3) and (4) are required to be scaled to concentration. In order to scale the amounts to concentrations an assessment of hydration status was conducted by the field investigator for both mother and child. In the absence of changes in status the mother’s volume of distribution is expected to be constant over the 14 days of the study. The infant’s volume of distribution ($V_{b}$), however, will change (usually increasing due to infant growth) during the study period. Growth of the infant’s volume of distribution over this period can be determined by the change in infant weight [given by Eqs. (A1.8) and (A1.9) in Appendix 1].

In this work, either the fraction of D_2_O that crosses via breastmilk and into the infant or the volume of distribution of the infant is not identifiable. Since the fraction and therefore quantity of water ingested by the infant during breastfeeding is the focus of this work it is therefore necessary to assume a value of $V_{b}$ for all infants based on a reference covariate such as weight or height. Based on prior work the volume has been assumed to be a function of weight [25], or the combination of weight and height [26]. This is addressed in Eq. (A1.9) in Appendix 1.

The final model consists of four parameters $k_{mm}$,$V_{m}$, $CL_{mb}$ and $CL_{bo}$. Of these, the parameters $CL_{mb}$ and $CL_{bo}$ are of primary importance to determine the non-breastmilk water intake.

### Statistical models

A standard three-stage hierarchical model was used. (Stage 1) the model for the data; (Stage 2) the model for heterogeneity between individuals; (Stage 3) the model for the priors. An additional part is also presented here about the statistical models to calculate $R_{s}$.

#### Stage 1: model for the data

7 $$y_{ij} \sim N(f\left( {\varvec{\theta}_{\varvec{i}} ,x_{ij} } \right), \sigma^{2} )$$

where $y_{ij}$ denotes the $j{th}$ observation for the $i{th}$ subject, $f\left( {\varvec{\theta}_{\varvec{i}} ,x_{ij} } \right)$ is the expected value of the data from the model prediction, $\varvec{\theta}_{\varvec{i}}$ is a vector (dimension $p \times 1$, where $p$ is the number of parameters) of individual parameter values for the *i*th individual, $x_{ij}$ is a sampling time (and other design variables such as dose), $N$ represents a normal distribution with (in this case) zero mean and standard deviation $\sigma$.

#### Stage 2: model for heterogeneity between individuals

The distribution of an individuals’ PK parameter vectors $\varvec{\theta}_{\varvec{i}}$ are shown,

8 $$\varvec{ln}(\varvec{\theta}_{\varvec{i}} )\sim N_{p} ({ \ln }(\varvec{\mu}),{\varvec{\Omega}}),and$$

9 $${\varvec{\Omega}}\sim Q_{p} (\varvec{\rho},{\mathbf{V}})$$

where $\varvec{\mu}$ is a vector of mean population pharmacokinetic parameters and ${\varvec{\Omega}}$ is the variance–covariance matrix of between subject random variability. $N_{p}$ represents a *p*-dimensional multivariate normal distribution.

$Q_{p}$ is the quadratic form using the column vector ${\mathbf{V}}$ as a diagonal matrix, $\varvec{\rho}$ is the LKJ correlation matrix, generating random correlation matrices based on vines and extended onion method [29]. $Q_{p}$ is equivalent with the calculation result of ${\mathbf{V\rho V}}$ (where V is diagonal), which provides the variance–covariance matrix for the fitted parameters. A detailed description about $\varvec{\rho}$ and ${\mathbf{V}}$ can be found in [12].

#### Stage 3: model for the priors

Priors for the analysis include: (1) priors for the parameters and, (2) priors for the known variables. In this work there are a number of known variables, that are usually considered to be constants in other work (for instance, the prior model for milk composition). In this work, they are considered as random variables with a known mean and variance.

The prior of the residual variance is:

10 $$\sigma \sim N(0, 1000)\;{\text{with}}\; \sigma > 0$$

Here $\sigma$ is sampled from a truncated normal distribution.

The prior for the vector of mean parameters, in this study, $\varvec{\mu}$, i.e. $CL_{mb}$, $CL_{bo}$, $k_{mm}$, and $V_{m}$, is given by a low information prior was assumed for all:

11 $${ \ln }(\varvec{\mu})\sim N(0, 1000)$$

The priors of the variance–covariance matrix ${\varvec{\Omega}}\sim Q_{p} (\varvec{\rho},{\mathbf{V}})$ is:

12 $$\varvec{\rho}\sim {\text{lkj}}\_{\text{corr}}(1)$$

13 $${\varvec{\uplambda}}_{{\mathbf{i}}} \sim N(0, 1000)\;{\text{with}}\;{\varvec{\uplambda}}_{{\mathbf{i}}} > 0,\;{\text{and}}\;{\mathbf{V}} = \varvec{\lambda I}_{\varvec{p}}$$

Here $I_{p}$ represents a $p \times p$ identity matrix, the parameter “1” in the ${\text{lkj}}\_{\text{corr}}$ function is the shape parameter. In this case “1” represents a bounded uniform distribution on the space of correlations, and ${\mathbf{V}}$ is from a truncated normal distribution.

#### Statistical models to calculate $R_{s}$

The primary objective of this study is to determine whether the mother is exclusively breastfeeding her infant. The ingested water intake rate from sources other than breastmilk (denoted $R_{s}$) is used as the metric to describe quantitatively the exclusivity of breastfeeding and a criterion value of this metric will be determined from this study from which EBF and non-EBF characteristics of this and future populations can be evaluated. This is a natural and appropriate choice since if the ingested water intake rate from other sources is zero, it indicates absolute EBF, i.e. the only ingested water source for the infant is from breastmilk. The model development process of calculating $R_{s}$ are outlined, and the relevant assumptions are described in Appendix 1.

It should be noted that the purpose of this study is to provide the best estimate of parameters at individual level (e.g. $R_{s}$, $CL_{mb}$ and $CL_{bo}$ etc.) and the parameters at population do not hold a particular significance.

### Model development

#### Model selection

Model selection was based on two criteria, Watanabe-Akaike information criterion (WAIC) and Leave One Out (LOO) Cross Validation. Individual subject Visual Predict Checks (iVPCs) were also used to evaluate the model. For the iVPCs all individual posterior samples (pooled across all chains) minus the burn-in samples were used.

The WAIC was defined as:

14 $${\text{WAIC}} = - 2\mathop \sum \limits_{j = 1}^{{N_{i} }} \mathop \sum \limits_{i = 1}^{n} { \log }\left( {\frac{1}{s}\mathop \sum \limits_{s = 1}^{S} p\left( {y_{i,j,s} |\theta_{i,s} } \right)} \right) + 2{\text{pWAIC}} ,$$

where $N_{i}$ is the number of observations for subject $i$, $n$ is the number of subjects; $y_{i,j,s}$ is the *s*th sample of the *j*th observation for the *i*th subject; $\theta_{i,s}$ is the *s*th sampled parameter for individual $i$ and $S$ is the number of samples. The first term on the right hand side of the equation is equivalent to the log density of the data, in − 2log(likelihood) form and the second term $2{\text{pWAIC}}$ represents a correction for the effective number of parameters to adjust for overfitting. To estimate the term $2{\text{pWAIC}}$, the method [37] computes the posterior variance of the log predictive density for each data point. Summing over all the data points gives the effective number of parameters as,

15 $${\text{pWAIC}} = \mathop \sum \limits_{j = 1}^{{N_{i} }} \mathop \sum \limits_{i = 1}^{n} V_{s = 1}^{S} ({ \log }p(y_{i,j,s} |\theta_{i,s} ))$$

Essentially WAIC is an extension of the Deviance Information Criteria (DIC). The DIC criteria is calculated at a point estimate of the parameters and may be unstable and slow to converge [38, 39]. Instead, WAIC is fully Bayesian and is based on computation over the full posterior.

LOO Cross Validation was also used in this study to evaluate the model performance. Vehtari et al. [40] proposed to use Pareto Smoothed Importance Sampling (PSIS), a new approach to compute LOO using importance weights. PSIS was used in this study since it has two advantages. First it provides additional stability on the calculation of LOO. Second, PSIS is able to approximate LOO with the already available posterior distributions from the full data which saves the computational time.

#### Bayesian analysis settings

Models were parameterized in terms of the natural log of the parameters values [41] (e.g. ln(CL), ln(*V*)). In the present study, three MCMC chains were run simultaneously. Each MCMC chain was run for 10,000 samples (excluding the 1000 samples that were discarded during the burn-in phase). The three MCMC chains were pooled to represent the posterior distributions of the parameter values of interest.

#### Model evaluation

The initial estimates of all the chains were selected by Stan randomly. Convergence of the MCMC chains were assessed using the potential scale reduction factor, Rhat (a measure of the ratio of between and within chain variability). All MCMC chains were assumed to have reached the stationary distribution if Rhat values were close to 1.0 for all parameters [42]. Furthermore, the trace history of MCMC samples for all chains were examined visually for all parameters, in which a ‘fuzzy caterpillar’ [15] suggests that MCMC chains had reached a stationary distribution. In addition, the number of effective samples in a chain “n_eff” [43] was used to investigate the sampling efficiency (i.e. the number of independent samples) during the analysis for computation of summary measures. In addition, visual predictive checks based upon individual mother–infant pair observations were available for evaluation.

### The criterion for the cut-off value of $\varvec{R}_{\varvec{s}}$ relating to EBF

The cut-off value of $R_{s}$ (water intake from non-breastmilk sources) to distinguish EBF and non-EBF was determined on the basis of the pooled individual posterior distributions of $R_{s}$. In the first step the individual posterior distributions of $R_{s}$ were determined for each mother–infant pair. Then the $R_{s}$ values for each study pair were pooled over all mother–infant pairs from the calibration data set to form a mixture distribution which was normalised by the total number of samples to yield a population density of $R_{s}$.

The cut-off value was elicited a priori based on expert opinion (AD, CS, TP, RG and LH). In this process the investigators indicated that they expected about 90% of the participants in the calibration study would have been exclusively breastfed, even given the rigorous field study techniques that were used. The cut-off value of the population density of $R_{s}$ was therefore set at 0.9 (i.e. the criterion yields 90% of the total area under the mixture distribution curve).

## Results

### Data

There were 121 mother–infant pairs recruited into this study. Seven pairs were identified by the field assistants during the study period as non-EBF due to the intake of water from a source other than breastmilk and were removed from further analysis. Consequently, the calibration data set consisted of 114 subjects with 1516 observations.

In this study, it is considered that data that arose from a mother–infant pair to be biologically implausible pair if *CL*_*bo*_ > 40% of infant body weight. For example, for a 5 kg infant the total water content would be about 3 kg and hence a value of water clearance of 2.0 kg/day is biologically incompatible with life. This exception resulted in one further mother–infant pair to be removed from the analysis. Ultimately, there were a total of 113 EBF mother–infant pairs with 1500 observations in the analysis. The demographics of the mother–infant pairs is presented in Table 1.

**Table 1:** Summary statistics of the mother–infant pairs included in the calibration study

Variable	Median (range)
Mother–infant pair no.	113
Dose (g)	30.0 (30.0–30.2)
Baby age (month)	3.3 (2.0–5.4)
Baby WT start (kg)	5.9 (3.9–8.4)
Baby WT end (kg)	6.2 (4.3–8.5)
Baby gender	54 (m)/59 (f)
Mother age (year)	25 (16–42)
Mother WT (kg)	53.1 (34.5–93.1)

### Final model

Different error models and covariates were tested and the model performance were evaluated quantitatively and graphically. The combined error model, mother’s weight on her volume of distribution ($V_{m}$) in Eq. (16) and baby’s weight on the clearance rate ($CL_{bo}$) in Eq. (17) were selected to be the full model (i.e. the best final model) because, (1) The combined error model was statistically preferred to the additive error model according to the WAIC and LOO values as the item (2) in Supplement 3, Table S3.1; (2) addition of mother’s weight on $V_{m}$; baby’s weight on $CL_{bo}$ also agree with the marginal correlations (see Supplement 3 Figs. S3.1 and S3.2) and also according to the biological plausibility. The covariate relationships were given by:

16 $$\ln \left( {V_{m, i} } \right) = N(3.49, 0.07) + N(0.62, 0.04)\ln \frac{{{\text{MWT}}_{\text{i}} }}{{70\;{\text{kg}}}}$$

17 $$\ln \left( {CL_{bo, i} } \right) = N( - 0.16, 0.17) + N(0.17, 0.03)\ln \frac{{{\text{BWT}}_{\text{i}} }}{{5\;{\text{kg}}}}$$

where $N$ is normal distribution with the distribution mean and standard deviation; MWT is mother’s weight; BWT is baby’s weight. 70 and 5 kg are the median value of mother’s and infant’s weights respectively. $i$ is the *i*th individual.

The details of the structural, error and covariate models are presented in the Stan code in Supplement 4.

### Parameter estimates and diagnostics for the fitting process

**Fig. 2:**
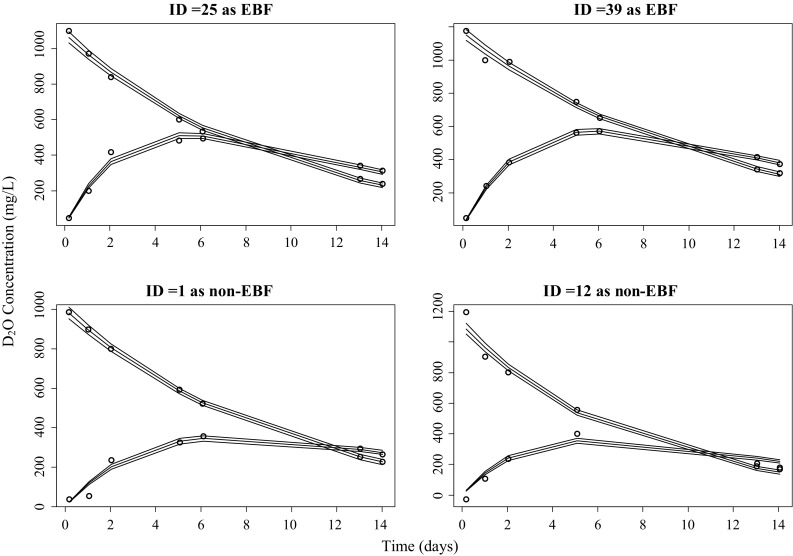
Individual Visual Predictive Checks for model evaluation. Open circles are the observations. The solid lines represent the median, 2.5% and 97.5% quantiles of the posterior distribution of the model predicted response. The upper curves represent the mother and lower curves the infant. ID = 25 and 39 are later classified as EBF. ID = 1 and 12 are later classified as non-EBF

The mean and 95th percent credible interval of each individual’s posterior distributions of the parameter values ($k_{mm}$,$V_{m}$, $CL_{mb}$ and $CL_{bo}$) and the calculated posterior distribution of $R_{s}$ are provided in Supplement 5. The population values of these parameters (i.e. population mean and between subject variability) are presented in Supplement 6. Sufficient samples need to be available from the pooled population posterior distribution of $R_{s}$ to ensure an accurate description of the 90th percentile in order to define the cut-off value for EBF. The sampling chains were superimposed and appeared to be well mixed, and the Rhat values were close to 1.0, indicating that a stationary solution was found. iVPC graphics for each mother–infant pair were plotted to evaluate the final model performance. iVPCs for all the pairs showed that the model describes the observations satisfactorily and four (2 later classified as EBF and 2 non-EBF) representative graphics are presented in Fig. 2.

A mother–infant pair can then be determined as EBF (or non-EBF) based on their posterior distribution of $R_{s}$, in combination with the *Rs* cut-off value determined below.

### $\varvec{R}_{\varvec{s}}$ cut-off value

**Fig. 3:**
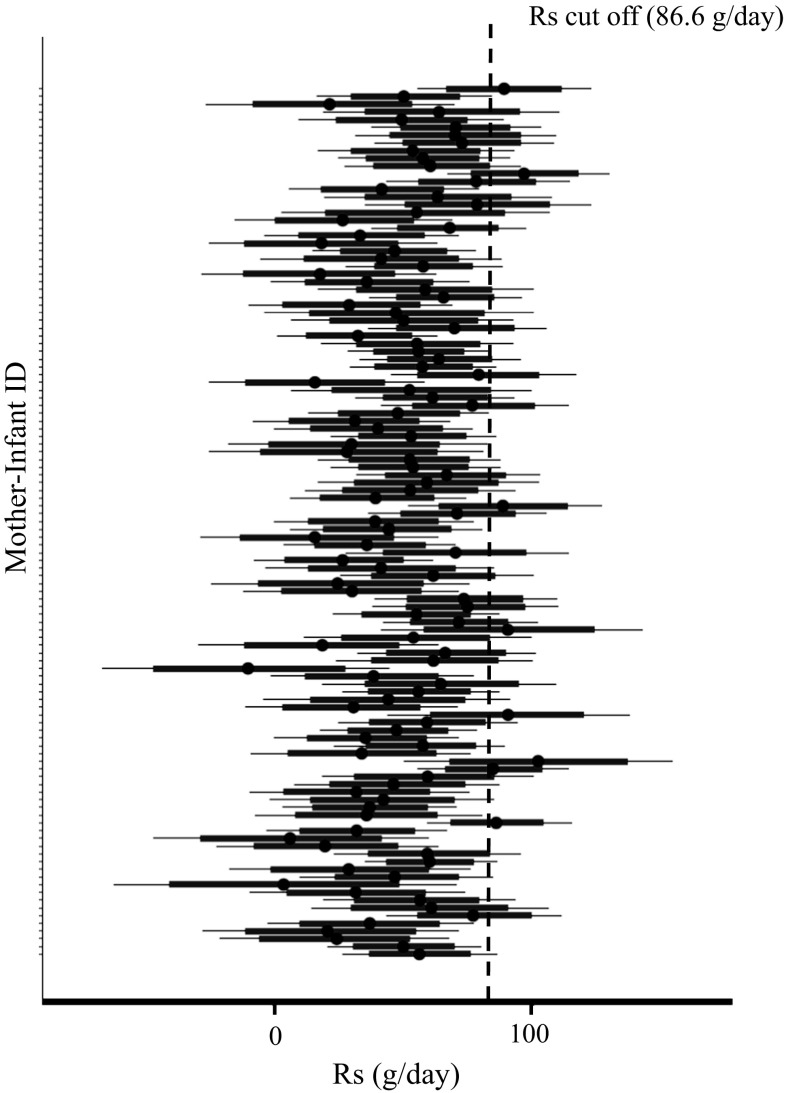
The individual posterior densities of $R_{s}$ and the $R_{s}$ cut-off value (at 86.6 g/day). Black dot is the mean of individual $R_{s}$ posterior distribution. Thick red line is the 25 and 75% quantiles and thin black line is the 2.5 and 97.5% quantiles (Color figure online)

**Fig. 4:**
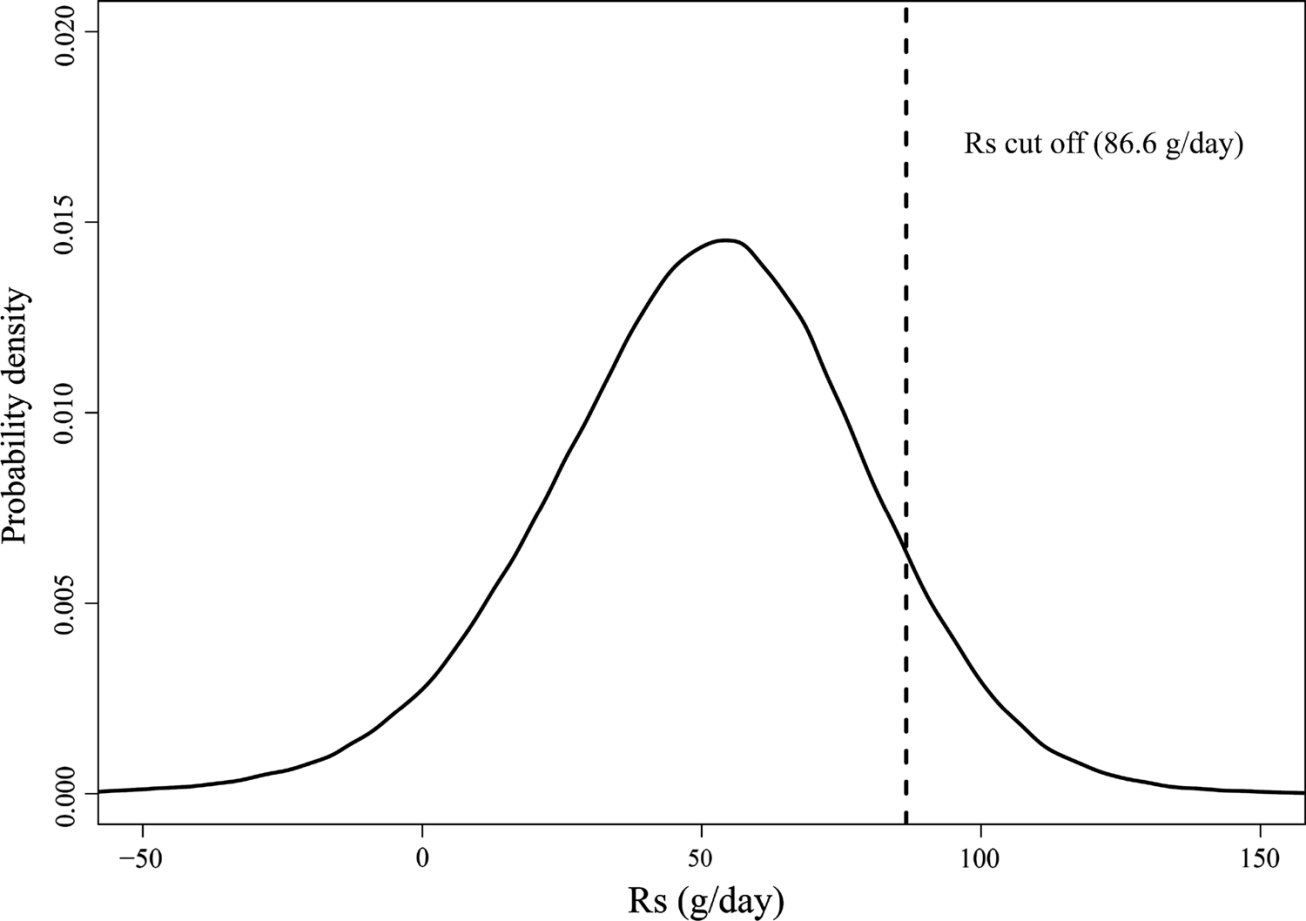
The pooled probability density function of $R_{s}$ and the identified cut-off value distinguishing EBF and non-EBF

The final model was used to determine the pooled posterior distribution of $R_{s}$ over all mother–infant pairs. The individual posterior densities are shown in Fig. 3 and the pooled density in Fig. 4. The $R_{s}$ cut-off value, determined as the 90th percentile of the pooled posterior distribution, was 86.6 g/day. This is similar to the value calculated from base model (the model without covariates) of 84.6 g/day.

## Discussion

In this work a hierarchical model describing the kinetics of deuterium in mother–infant pairs was described. This model included uncertainty in the population parameters (at the prior level) as well as uncertainty in the constants that were not able to be estimated from the available data. The final model showed that the concentration–time profiles could be estimated with acceptable accuracy. From this it was possible to determine a value of $R_{s}$ (infant water intake (g/day) from non-milk sources) that could be used as a criterion for classification of the practice of mother–infant pairs as exclusively or non-exclusively breastfeed.

In this study, the mothers were instructed to maintain EBF practice and more importantly, were monitored by field assistants. To our knowledge, this is the first study that has used a direct and objective monitoring method to assess the mother’s breastfeeding practice. These rigorous field study techniques are likely to be superior to the traditionally used methods (e.g. caregiver recall) which are subjective and may be biased [9]. It is believed therefore, that the recruited mother–infant pairs in this study could be considered as representative of best EBF practice. However it is expected even in this best-practice population that some mother–infant pairs may not have been perfectly EBF and therefore, based on expert opinion, the cut-off criterion was based on the belief that 90% of samples of $R_{s}$ were likely to be EBF. Hence it is possible that our EBF cut-off criteria may be conservative and the actual cut-off value being greater than 86.6 g/day. However, it is noted that our cut-off value of $R_{s}$ of 86.6 g/day is considerably higher than previously suggested at 10–25 g/day. In order to assess the relevance of the previous value in relation to the experimental design used for DTM studies a theoretical lower limit of the value of $R_{s}$ was calculated. This lower limit was determined as the 95% upper bound of the posterior distribution of $R_{s}$ for a mother–infant pair where the mother was a theoretical perfect EBF (i.e. where the true $R_{s}$ was set to zero). The posterior distribution in this case accounts for the uncertainty in the parameter values and fixed constants associated with DTM analysis technique recommended by IAEA, International Atomic Energy Agency [11]. The cut-off value was determined to be 56 g/day (see Supplement 7 for relevant calculations). An interpretation of this value would indicate that it is impossible to distinguish 56 g/day from 0 g/day by using the D_2_O DTM technique irrespective of how rigorous the field experiment or how fastidious the mother. The $R_{s}$ cut-off (86.6 g/day) identified in this work was interpreted as the sum of the theoretical $R_{s}$ lowest limit (56 g/day) and experimental error introduced in the field work that propagates into the analysis. In the work of Haisma et al. [25], the mean EBF $R_{s}$ value was reported as 10 g/day. Moore et al. [7] reported the $R_{s}$ distribution in their EBF group as 24.6 ± 62.1 g/day (with 24.6 g/day as the cut-off value). We believe this value of $R_{s}$ is essentially impossible replicate in any DTM field study and would result in almost all mother–infant pairs as non-EBF despite their best practice.

The purpose of applying a full MCMC approach in this work was to allow full enumeration of the uncertainty in the parameters of the kinetic model that accounts for uncertainty in all unknown “constants”. From these posterior distributions the distribution region of normal deuterium exposure in mother–infant pairs related to EBF (i.e. $R_{s}$ posterior distribution) was computed, and the $R_{s}$ cut-off criterion distinguishing EBF from non-EBF was also determined. By using this criterion in combination with the $R_{s}$ posterior distribution, rather than assign a new mother–infant pair as EBF or non-EBF their posterior probability that the pair were EBF can be computed and estimation for uncertainty and subjectivity in the inference from future field studies is possible.

Even though attempts were made to account for uncertainty in the many constants that contribute to the D_2_O DTM approach, it is difficult to accommodate fully for these error sources. For estimation of the $R_{s}$ posterior distribution to assess the EBF practice, appropriate calculation of the water through atmospheric water absorption (i.e. $R_{a}$) is necessary. In this work, it is assumed that 6.3% (SE 1.7%) of the total water input was from the atmospheric water absorption. This value was based upon the experiment conducted in Cambridge, UK [26]. However, the mother–infant pairs and the experiment conducted in our study were in Sumedang, Indonesia. The climate in these two regions differ significantly, in terms of temperature, moisture etc. (e.g. a range of ambient temperature in Cambridge, 4–18 °C and in Sumedang, 28–31 °C). It is speculated that the atmospheric water absorption percentage in Indonesia differs from the UK. This difference might cause a noticeable change in the final $R_{s}$ cut-off value. Due to the unavailability of the atmospheric water absorption percentage in Indonesia, it was assumed that the percentage in the UK is also applicable in our study. More generally speaking, the $R_{s}$ cut-off value is likely to be geographically and climatically dependent, meaning, different regions might have different $R_{s}$ cut-off values based upon the local climates. It is however evident that determination of $R_{s}$ cut-off value in different regions could be challenging. On the other hand, the ultimate goal of this study is to categorize the new mother–infant pairs into EBF or non-EBF groups based on the determined $R_{s}$ cut-off value and promote those non-EBF mothers to EBF. It is believed that part of the inflation of our $R_{s}$ cut off at 86.6 g/day (over and above the theoretical 56 g/day) will reflect systematic bias in its estimate due to $R_{a}$ and possibly other factors. We do not believe that this will result in misclassification of mother–infant pairs in similarly humid regions but perhaps this cut-off may be less conservative in more temperate regions.

The $R_{s}$ cut-off value was determined based on expert opinion that 90% of the participants in the calibration study had been exclusively breastfed and accordingly the cut-off value yields 90% of the total area under the probability density curve. The value of 90% reflects of the confidence about all the recruited mother–infant pairs being EBF, which is ultimately a subjective judgement. On the other hand, the judgement was supported by the rigorous field study techniques that were used (e.g. the direct monitoring methods). The application of these techniques is more superior and reliable than, e.g. the biased caregiver recall method.

It is intended that this work is used to help low-income countries identify health burden risk associated with breast feeding practice in their particular regions. A cut-off value for $R_{s}$ therefore provides a critical measure which can help regions identify at risk populations and therefore target areas where appropriate public health measures may need to be introduced. The next stage of this global health project is to identify a simpler DTM design that could be conducted more readily by field workers across many diverse regions.

## Conclusions

A nonlinear hierarchical model within a Bayesian framework was successfully developed for the description of deuterium oxide kinetics in exclusively breastfeeding mother–infant pairs. A cut-off value for a biomarker was determined that could be used for distinguishing the exclusivity of breastfeeding practice. The cut-off could be used to categorize any new cohort of mother–infant pairs as EBF or non-EBF group.

### Electronic supplementary material

Below is the link to the electronic supplementary material.

Supplementary material 1 (DOCX 14 kb)

Supplementary material 2 (DOCX 94 kb)

Supplementary material 3 (DOCX 20 kb)

Supplementary material 4 (DOCX 16 kb)

Supplementary material 5 (XLSX 40 kb)

Supplementary material 6 (DOCX 12 kb)

Supplementary material 7 (DOCX 12 kb)

## Figures and Tables

**Fig. 5:**
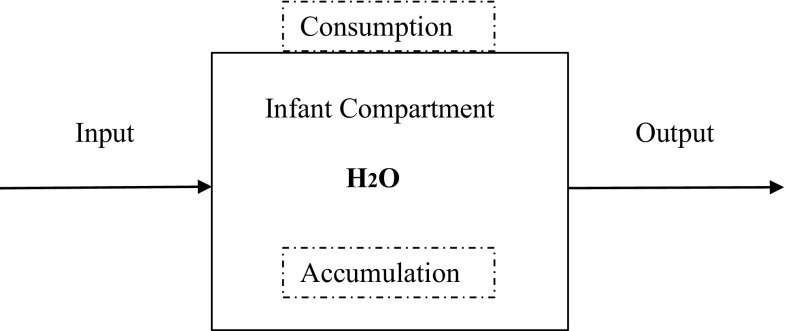
Mass balance model structure of water in infant’s compartment

## Appendix 1: $\varvec{R}_{\varvec{s}}$ model development

To calculate the $R_{s}$, we need to consider the mass balance of water for the infant. The schematic in Fig. 5 represents the mass balance model structure for infant compartment on total water.

According to the rule of mass balance,

A1.1 $$Accumulation = input - output - water\;for\;growth$$

In terms of water (w), we have,

A1.2 $$dw/dt = (CL_{mb} + R_{m} + R_{s} + R_{a} ) - \left( {R_{{c\left( {bo} \right)}} + R_{g} } \right)$$

where $dw/dt$ is the rate of accumulation of water; $R_{m}$ is the intake rate of water metabolised from protein, fat, and carbohydrate in breastmilk (units: L/day); $R_{s}$ is the ingested water intake rate from sources other than breastmilk (units: L/day); $R_{a}$ is the water intake rate from absorption of atmospheric water by lungs and skin (units: L/day); $(CL_{mb} + R_{m} + R_{s} + R_{a} )$ is the total water input rate to the infant (units: L/day); $R_{{c\left( {bo} \right)}}$ is the total water output from the infant, i.e. flow from the infant to the outside, which includes water lost as urine, sweat, in faeces and in breath, including a correction for isotopic fractionation (discussed in the next subsection) (units: L/day); $R_{g}$: is the water that is converted by the infant into permanent tissues due to growth (units: L/day).

At this stage, we consider that no water is accumulated in the infant over than by growth (see Assumption 1).

### Assumption 1

The infant’s compartment is considered in steady state. Total flow into the compartment is equal to total flow out of the compartment.

Therefore, we can rearrange Eq. (A1.2) to solve for the water intake from sources other than breastmilk.

A1.3 $$R_{s} = R_{c(bo)} + R_{g} - CL_{mb} - R_{m} - R_{a}$$

The statistical models used to calculate $R_{c(bo)}$, $R_{g}$, $R_{m}$ and $R_{a}$ are presented as below. NB, the value of $CL_{mb}$ is part of the structural model for which the posterior distribution is estimated.

### $R_{m}$: intake rate of water metabolised from protein, fat, and carbohydrate in breastmilk

Human breastmilk comprises free water, protein, fat, and carbohydrate. Water is also produced in the infant by metabolising the protein, fat, and carbohydrate contained in breastmilk. In order to calculate the metabolised water intake rate ($R_{m}$, units: L/day), we need to know, (1) the human milk composition (i.e. the proportion of free water, protein, fat, and carbohydrate in human milk), (2) the total mass of breastmilk intake per day ($M$, units: kg/day), and (3) the mass of water produced by metabolising, e.g. 1 g of protein in human milk.

Prior information is used on the human milk composition. Gidrewicz and Fenton [30] conducted a systematic review and meta-analysis of the nutrient content of preterm and term breastmilk, with all values for fat limited to 24-h breastmilk collections. Studies conducted in low income countries were excluded. As the infants recruited in our study were full-term (37–42 weeks gestation), the composition of full-term breastmilk was used in the analysis. Notice here that the breastmilk composition varies with prematurity and postnatal age [30]. The breastmilk composition applied in this study is based on data from day 4 to week 12 of lactation and are presented in Table 2.

**Table 2:** The weighted mean and standard error (SE) for human milk composition (g/100 g human milk)

	Protein	Fat	Lactose	Free water
Weighted mean	1.0	3.4	6.7	88.9
SE of weighted mean	0.007	0.092	0.092	0.13

The data values are from the meta-analysis of the nutrient content of breastmilk reported by Gidrewicz and Fenton [30]

To calculate the total breastmilk intake mass $M$, we use $CL_{mb}$ divided by free water proportion in breastmilk (see Assumption 2), which in this case is 0.889 (SE = 0.0013).

#### Assumption 2

The generally assumed water content of milk is sufficiently precise and accurate to estimate $M$, consequently to allow for an unbiased and precise estimate of $R_{s}$.

The formula to calculate $M$ is given in Eq. (A1.4). Here we apply a density of water of 1 kg/L.

A1.4 $$M\;({\text{kg}}/{\text{day}}) = CL_{mb} /\left[ {0.889 ({\text{SE}}, 0.0013)} \right]$$

The yield of water from 1 g of protein is 0.41 g [31], from 1 g of fat 1.07 g and from 1 g of carbohydrate 0.55 g [32]. According to Table 2, 100 g breastmilk contain 1.0 g protein (SE, 0.007 g), 3.4 g fat (SE, 0.092 g) and 6.7 g lactose (SE, 0.092 g). Therefore, water produced in the infant from the oxidation of protein, fat, and carbohydrate in 100 g breastmilk is as presented in Eqs. (A1.5) and (A1.6). The variable $W.F.O$ represents water from oxidation.

A1.5 $$W.F.O\;(mean) = 1.0 \times 0.41 + 3.4 \times 1.07 + 6.7 \times 0.55 = 7.733\;{\text{g}}$$

A1.6 $$W.F.O\;(SE) = (0.007^{2} \times 0.41 + 0.092^{2} \times 1.07 + 0.092^{2} \times 0.55)^{0.5} = 0.117\;{\text{g}}$$

This indicates 100 g breastmilk produces 7.733 g water in average by oxidation process. Consequently, the metabolised water intake rate ($R_{m}$) to the infant derived from breastmilk is given by Eq. (A1.7) (see Assumption 3). Notice here $R_{m}$ is in the unit of L/day.

A1.7 $$R_{m} (L/day) = [0.07733 ({\text{SE}}, 0.00117)] \times M$$

#### Assumption 3

That the generally assumed model for $R_{m}$ is sufficiently precise and accurate to estimate $R_{m}$, consequently to allow for an unbiased and precise estimate of $R_{s}$.

### $R_{g}$: Water retaining rate for infant’s growth

The total body water ($TBW$) increases due to the infant’s growth during the experimental sampling period. A portion of water (shown as the “Water for growth” component in Eq. (A1.1) is retained in the infant’s body contributing to the infant’s growth. The relationship between the increased $TBW$ and the water retained due to the growth rate $R_{g}$ is:

A1.8 $$R_{g} (L/day) = (TBW_{ls} - TBW_{fs} )/(day_{ls} - day_{fs} )$$

$TBW_{ls}$ and $TBW_{fs}$ represent the infant’s total body water at the last sampling day (i.e. subscript *ls*) and at the first sampling day (i.e. subscript *fs*), respectively and $day_{ls} - day_{fs}$ describes the experimental sampling period, usually 14 days in this study.

$TBW$ is calculated with Eq. (A1.9) [33], according to the infant’s weight.

A1.9 $$lnTBW (L) = a + \left( {b \times lnWT} \right)$$

where $$a \sim N( - 0.427, 0.012)$$, $$b \sim N(0.963, 0.005)$$.

In this study, the infant’s weight was measured at the first sampling day ($$day_{fs} )$$ and the last sampling day ($$day_{ls} )$$. $$TBW_{ls}$$ and $$TBW_{fs}$$ can be calculated with Eq. (A1.9). Thus $$R_{g}$$ can be calculated with Eq. (A1.8) (see Assumption 4).

#### Assumption 4

That the current equations for $TBW$ are sufficient to describe $R_{g}$ and provide an unbiased and precise estimate of $R_{s}$.

Since neither the volume of distribution of the infant ($V_{b}$), nor the fraction of D_2_O that cross via breastmilk are identifiable, calculation of $V_{b}$ based on prior work is needed. In this study, $V_{b}$ is calculated based on the infant’s $TBW$. To do so, correction for non-aqueous isotopic exchange is necessary and $V_{b}$ is assumed to be 4.1% larger than $TBW$ [11]. Therefore, the infant’s volume of distribution is calculated with Eq. (A1.10) at $day_{fs}$ and at $day_{ls}$. $V_{b}$ is assumed to change linearly with time due to growth, thus $V_{b}$ at other sampling times can be calculated (see Assumption 5).

A1.10 $$V_{b} (L) = 1.041 \times TBW$$

#### Assumption 5

That the current equation and method for calculating $V_{b}$ are sufficient to provide an unbiased and precise estimate of $R_{s}$.

### $R_{c(bo)}$: total infant’s water output rate after isotopic fractionation correction

The D_2_O DTM technique uses deuterium as a tracer for describing water kinetics. Since D_2_O is not identical to H_2_O with respect to its physical properties, deuterium is lost from body water via breath and insensible routes via the skin (transdermal evaporation) more slowly than light water. This phenomenon is called isotopic fractionation. Therefore, a rectification for non-equivalence of insensible loss of heavy vs light water is needed. Total water output from the infant, i.e. flow from the infant to the outside ($CL_{bo}$) must be corrected for isotopic fractionation. The results for the hydrogen isotope fractionation factor $\alpha_{1 - v} \left( D \right)$ from most of the literature were regressed to the equation [34] as:

A1.11 $$10^{3} \ln n\alpha_{1 - v} \left( D \right) = 1158.8\left( {\frac{{T^{3} }}{{10^{9} }}} \right) - 1620.1.8\left( {\frac{{T^{2} }}{{10^{6} }}} \right) + 794.84\left( {\frac{T}{{10^{3} }}} \right) - 161.04 + 2.9992\left( {\frac{{10^{9} }}{{T^{3} }}} \right)$$

Equation (A1.11) is described as valid from the freezing temperature of water (273.15 K) to the critical temperature (647.25 K) within ± 1.2(1σ) (n = 157); *T* is temperature in Kelvin (K).

A healthy infant’s rectal temperature is considered between 36.6 and 38 °C from various medical information guidance sources [35, 36]. According to Eq. (A1.11), the isotopic fractionation factor for deuterium between water vapour and water liquid is between 0.937 at 36.6 °C and 0.938 at 38 °C. Since the infant’s rectal temperature is unknown in this study, we approximate the mean fractionation factor is distributed uniformly over the range [0.937–0.938]. The fractionation rate of infant’s water output has been estimated [25] where 85% of the water output is not fractionated and that the remaining 15% is fractionated. The correction factor is therefore 0.85 + (0.937–0.938) × 0.15, which is 0.99055–0.9907. Due to the narrow range of the correction factor, it is treated as a constant 0.9906, $R_{{c\left( {bo} \right)}}$, and is shown in Eq. (A1.12) (see Assumption 6).

A1.12 $$R_{{c\left( {bo} \right)}} (L/day) = CL_{bo} /0.9906$$

#### Assumption 6

That the current rectification method for differentiating insensible loss of deuterium compared to light water can be characterised by Eq. (A1.12) and is sufficient to provide an unbiased and precise estimate of $R_{s}$.

### $R_{a}$: Absorption of atmospheric water by lungs and skin

The final component in the calculation of $R_{s}$ requires accounting for the water intake rate from absorption of atmospheric water by the lungs and skin, as $R_{a}$. It has been found that the water intake by absorption is proportional to the total water intake, and in that work [26] 21 healthy full-term formula milk fed infants in Cambridge, UK, at 12 weeks of age provided data on both total water intake and water absorption. Summary data are provided in Table 3.

**Table 3:** Reported non-oral water intake proportion by absorption

Mean	SE	Samples	Reference
0.063	0.017	21	[26]

As the total water input is equal to total water output $R_{{c\left( {bo} \right)}}$ plus water for growth $R_{g}$, $R_{a}$ is given by Eq. (A1.13) (see Assumption 7).

A1.13 $$R_{a} (L/day) = \left[ {0.063 \left( {{\text{SE}}, 0.017} \right)} \right] \times \left( {R_{{c\left( {bo} \right)}} + R_{g} } \right)$$

#### Assumption 7

That the estimate of the proportion of non-oral water intake can be accurately characterised by Eq. (A1.13) and is sufficient to provide an unbiased and precise estimate of $R_{s}$.
