# Supplementary material for: Development of a nonlinear hierarchical model to describe the disposition of deuterium in mother–infant pairs to assess exclusive breastfeeding practice
Source: J Pharmacokinet Pharmacodyn. 2018 Nov 14;46(1):1–13. doi: 10.1007/s10928-018-9613-x (PMC6394541; doi:10.1007/s10928-018-9613-x)
Supplement: Supplementary file 1 — Supplementary material 1 (DOCX 14 kb) [file 10928_2018_9613_MOESM1_ESM.docx]

**Supplement 1: Analysis of averaging the sampling times and concentrations.**

In the original calibration data set, one sample was collected on each of days 0,1,2,5,6,13 and 14. An additional sample was collected on each of days 0, 14, 5 or 6. The collection time of the additional sample on these days was within 1 hour of the original sample. In this study, the model for the data is presented briefly here in equation (S1.1) and can be also seen in details in the section of Statistical model. The implicit assumption in this equation is the samples are independent.

$y_{ij} \sim N(f\left( \boldsymbol{\theta}_{\boldsymbol{i}},x_{ij} \right), \sigma^{2})$ (S1.1)

The difference of the observed ($y_{ij}$) from the model predicted ($f_{ij}$) is assumed to be uncorrelated over time in order to claim independence, a necessary assumption in the modelling process. However, the two samples collected in one hour on the same day (day 0, 14, 5 or 6), may violate the assumption of independence and affect the parameter estimation process. To accommodate this issue, the concentrations from the two sampling times were treated as repeated measures and averaged for mother and infant separately. Since this increases the information content in the averaged repeated measures an adjusted residual error model was incorporated:

(1) $y_{ij} \sim N(f\left( \boldsymbol{\theta}_{\boldsymbol{i}},x_{ij} \right), \sigma^{2})$, where $Var\left( e_{ij} \right)=\sigma^{2}$

(2) $y_{ij} \sim N(f\left( \boldsymbol{\theta}_{\boldsymbol{i}},x_{ij} \right), \sigma^{2}\beta^{z})$, where z = 0 for the single measure; z=1 for the averaged duplicate measures within 1 hour; β is uniform distribution 0 ≤ β ≤1

Incorporation of the more complicated residual error structure in (2) above allows the model to accommodate the additional information present in the averaged repeated measures. The value of β describes the divergence away from the anticipated equal weighting (β=1).

It is shown in Table S1.1, that WAIC and LOO are very close to each other in (1) and (2). In addition, β is also very close to 1. Consequently, (1) and (2) are essentially indistinguishable and the method of averaging the sampling times and concentrations at the same day does not significantly affect the fitting process.

Table S1.1. Analysis of the influence on averaging the sampling times and concentrations at the same day. The numbers in brackets are the standard deviations of the calculated WAIC and LOO.

|  | (1) $y=N(f, \sigma^{2})$ | (2) $y=N(f, \sigma^{2}\cdot\beta^{z})$ |
| --- | --- | --- |
| WAIC | 12973 (140.8) | 12972.8 (143.2) |
| LOO | 13013.6 (143.0) | 13019.2 (146.0) |
| β | - | 0.9 (0.04) |

All subsequent analyses used the simpler error model as (1), excluding the estimation of β.
