# Supplementary material for: Development of a nonlinear hierarchical model to describe the disposition of deuterium in mother–infant pairs to assess exclusive breastfeeding practice
Source: J Pharmacokinet Pharmacodyn. 2018 Nov 14;46(1):1–13. doi: 10.1007/s10928-018-9613-x (PMC6394541; doi:10.1007/s10928-018-9613-x)
Supplement: Supplementary file 2 — Supplementary material 2 (DOCX 94 kb) [file 10928_2018_9613_MOESM2_ESM.docx]

**Supplement 2. Structural model development**

The structural model used in this work was based on prior information about mass balance of water in humans and the currently accepted model used empirically for DTM D_2_O analysis. This is outlined briefly in the steps below. A number of assumptions are required in this analysis due to the required simplification from the standard model and relating to ^2^H compared to ^1^H.

In this work the base model was determined *a priori* (as indicated below). Covariates were explored for development of the final model in order to refine predictions of the clearance of D_2_O from mother to infant and from the infant. Initial covariate selection was based on visually assessing the relationship between the individual mean posterior estimates of the parameters vs the covariates. Covariates without obvious multicollinearity were considered initially. In addition, only those covariates with mechanistic plausibility were considered in the full covariate model. The covariates tested include country effects and phenotypic covariates.

*Step 1: General compartmental model describing D_2_O disposition*

It is generally accepted that water disposition can be approximated by a simple three-compartment model [19], in which water is present in three spaces, vascular, fast and slow distribution tissues. Since the flux of D_2_O is kinetically indistinguishable from water [20] and we assume that a mixture of diluted D_2_O in H_2_O has the same kinetic characteristics as standard H_2_O (See Assumption 1). This three-compartment model is presented in Figure S2.1. In this model fast tissues typically represent extracellular fluid (e.g. saliva, breastmilk etc.) and slow tissues typically intracellular fluid.

**Assumption 1**: D_2_O has the same disposition kinetics as H_2_O.


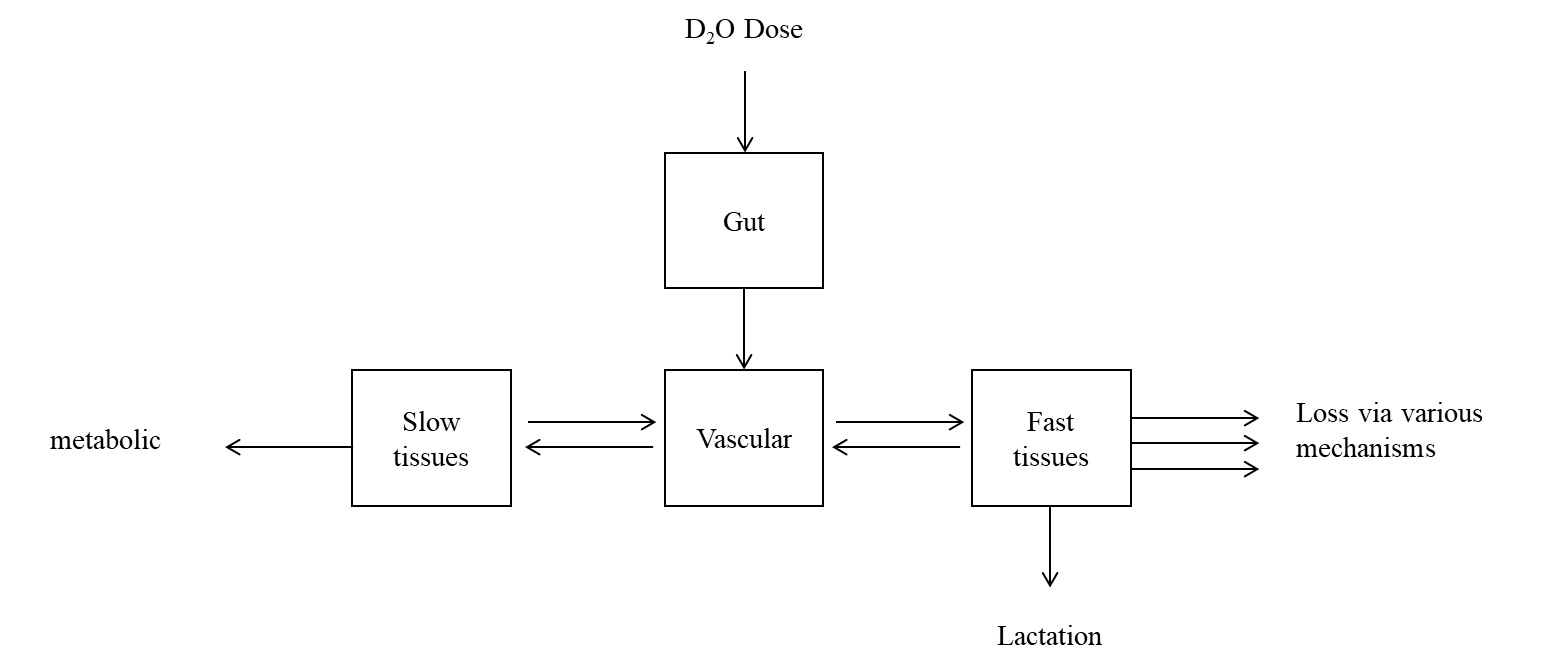


Figure S2.1: Schematic of D_2_O disposition (Adapted from Maguire [19]).

*Step 2: Incorporating the infant into the standard model*

An extended version of this model that includes the mother and the infant is shown in Figure S2.2. In this model, we divide the “Fast tissues” in the mother to include compartments for “Breastmilk”, “Saliva” (the sampling compartment) and “Other fast tissues (m)”. This allows study samples to be collected from the differentiated compartments. Similarly, we present the “Fast tissues” in the infant as “Saliva” and “Other fast tissues (b)”, because samples are only collected from the infant’s saliva.


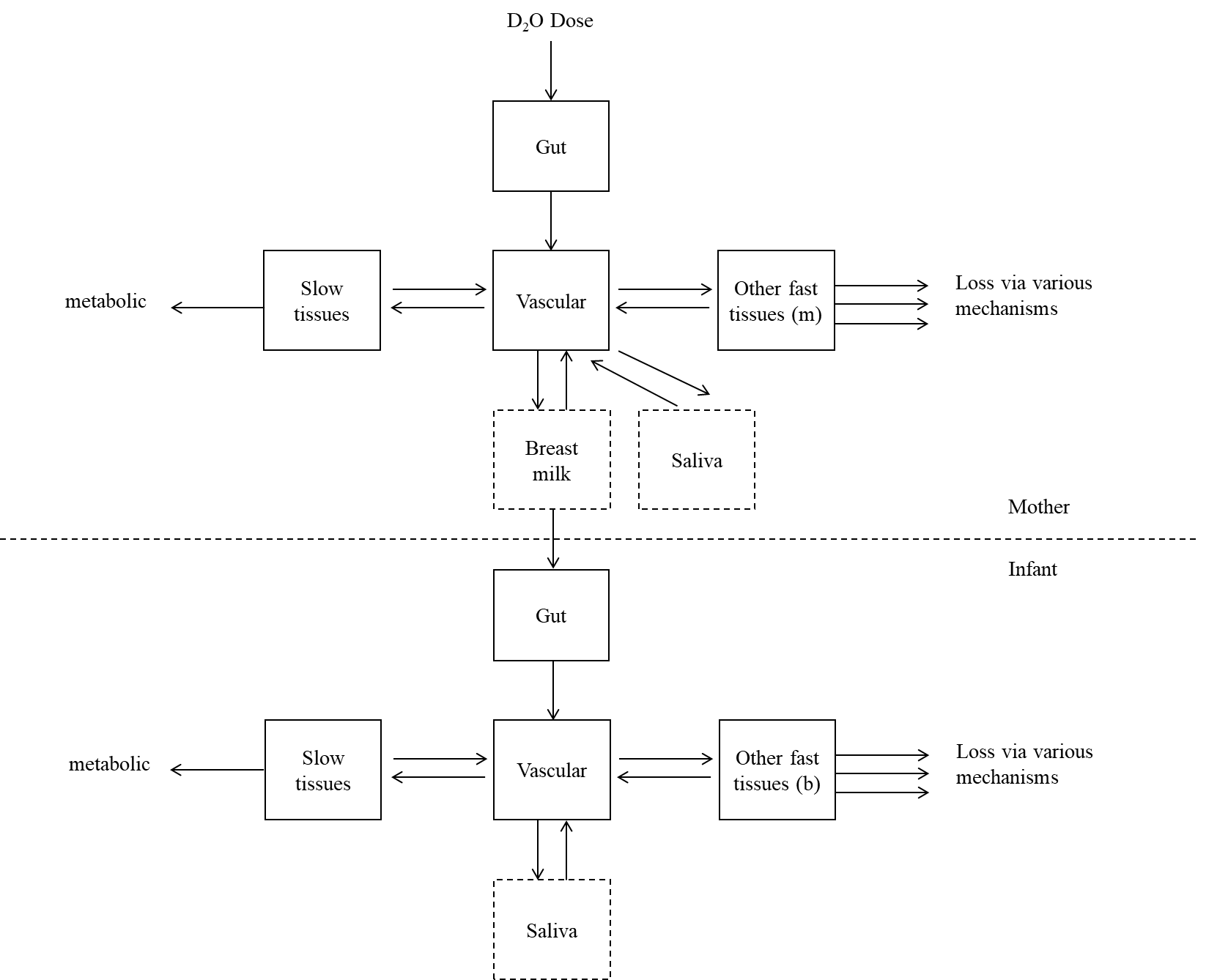


Figure S2.2: Schematic of D_2_O disposition in mother and infant; D_2_O transfers between mother and infant via breastfeeding; The broken-line compartments represent potential sampling sites.

*Step 3: Final mass transfer compartment model of breastfeeding*

It is assumed that the flux of water from vascular to fast tissues is much faster than changes in saliva concentrations and therefore it was considered unlikely to influence the saliva profile. Therefore, the mother’s compartments, including “Vascular”, “Other fast tissues (m)”, “Breastmilk” and “Saliva” can be lumped together. The infant’s compartments, including “Vascular”, “Other fast tissues (b)” and “Saliva” can be merged together as well (See Assumption 2).

**Assumption 2**: The distribution of D_2_O from the vascular space to fast tissues is extremely rapid with respect to the concentration-time profile in saliva, and these compartments can be lumped.

A simplified version of the model in Figure S2.2 based on Assumption 2 is shown in Figure S2.3. Of note, a similar two compartment model for water was proposed and reported for mother and infant respectively [21, 22]. Notice here the sample concentration of D_2_O is considered the same as the D_2_O concentration in the “Fast tissue” compartment (See Assumptions 3 and 4). Note if both saliva and breastmilk samples are available then a breastmilk compartment will need to be added as per Figure S2.2 to differentiate the two samples.

**Assumption 3:** The saliva concentration of D_2_O is representative of the Fast tissue compartment.

**Assumption 4:** The Fast tissue compartment is representative of the breastmilk compartment. This assumption can be relaxed if breastmilk samples are available.


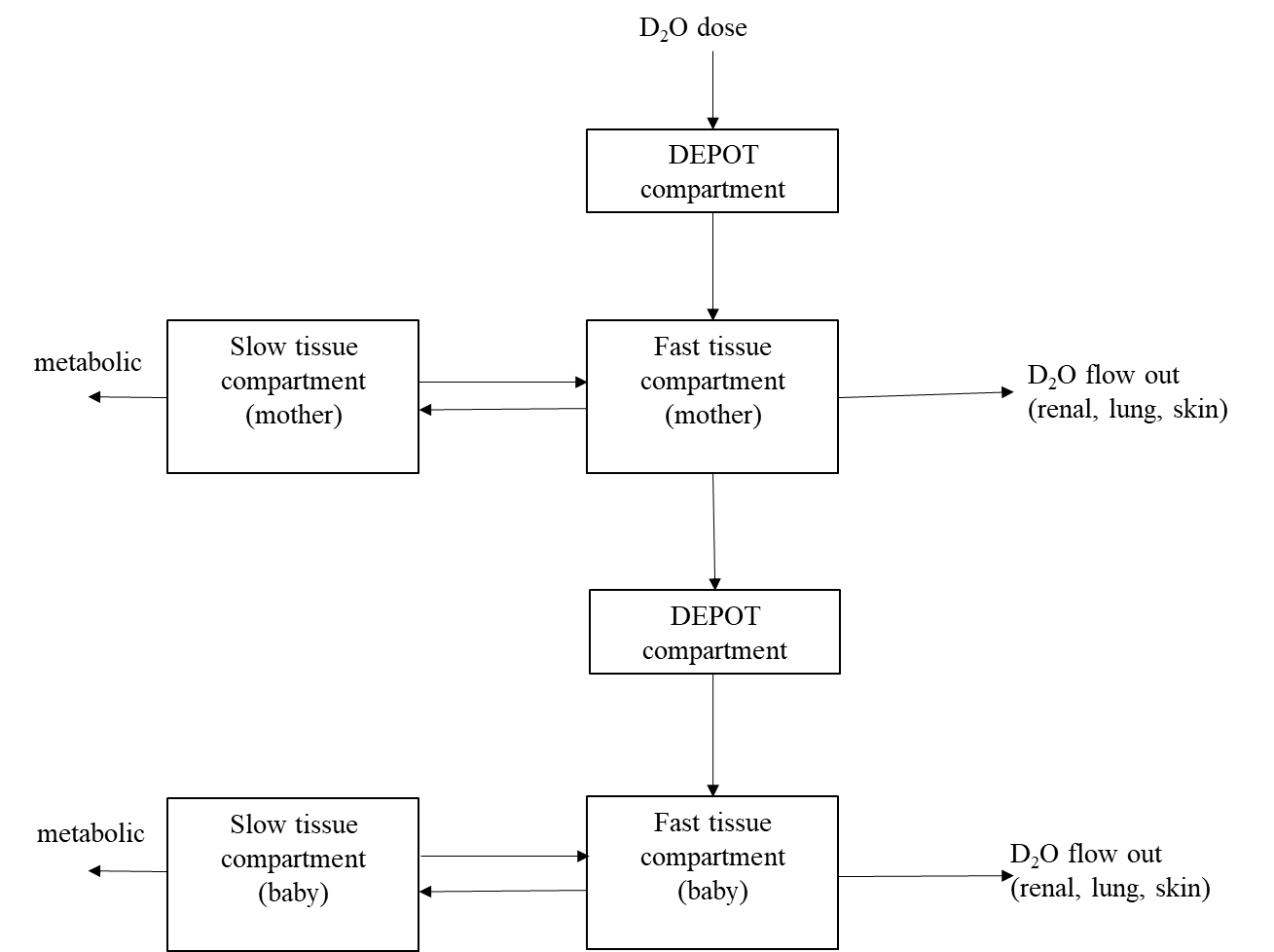


Figure S2.3: Schematic of D_2_O disposition in mother and infant, with concatenated vascular space and fast tissues into Fast tissue compartment

It is noted that the first sample taken for determining DTM D_2_O kinetics was taken > 3 hours post-dose to the mother. At this point all fast equilibrium has been complete and the remaining disposition is distribution limited from slow tissue compartments.

It is generally believed that water transport across cell membranes is essentially a near instantaneous process owing to the high hydraulic permeability of the cell membrane [23, 24]. Since D_2_O is kinetically indistinguishable from water (See Assumption 1), it is also believed that the D_2_O transport across cell membranes is not distinguishable from fast tissue sampling (See Assumption 5). Therefore, the Fast and Slow tissue compartments can be merged together. We assessed the influence of this assumption with simulation in which we determined whether samples taken after 3 hours would influence estimation of the lumped parameters. This was performed by simulating under the full model and then fitting the simpler model based on standard parameter values. No apparent bias in CL of water was seen. Additional assumptions included: the contribution of metabolic consumption of D_2_O was considered negligible and ignored (Assumption 6); and the gut (depot compartment in Figure S2.3) and fast tissue compartments can be lumped (Assumption 7).

**Assumption 5**: The distribution of D_2_O across fast and slow compartments is indistinguishable with respect to saliva sampling and these compartments can be lumped.

**Assumption 6**: The metabolic consumption of D_2_O in both mother and infant is small and can be ignored.

**Assumption 7**: The absorption of D_2_O from the gut is almost instantaneous and therefore the gut and fast-tissue compartments can be lumped.

The final model is represented by two linked 1-compartment disposition models that represent mother and infant, as in Figure 1 of the main text.
