# Supplementary material for: Development of a nonlinear hierarchical model to describe the disposition of deuterium in mother–infant pairs to assess exclusive breastfeeding practice
Source: J Pharmacokinet Pharmacodyn. 2018 Nov 14;46(1):1–13. doi: 10.1007/s10928-018-9613-x (PMC6394541; doi:10.1007/s10928-018-9613-x)
Supplement: Supplementary file 3 — Supplementary material 3 (DOCX 20 kb) [file 10928_2018_9613_MOESM3_ESM.docx]

**Supplement 3**

Table S3.1 presents the WAIC and LOO values for model diagnostics. Item (2) is the best base model (i.e. model without covariates). Item (3) is the best full model (i.e. model with covariates).

Table S3.1. WAIC and LOO values for model diagnostics. The smaller values of WAIC and LOO indicate better model descriptive performance. CEM = combined error model, with $V_{m}$~ MWT indicating inclusion of MWT (mother’s weight) as a covariate on $V_{m}$ and ${CL}_{bo}$ ~ BWT indicating inclusion of BWT (baby’s weight) as a covariate on ${CL}_{bo}$.

| **Error Model** | **LOO** | **WAIC** |
| --- | --- | --- |
| 1. Additive Error Model | 13233.8 | 13178.4 |
| 2. Combined Error Model | 12846.8 | 12803.2 |
| 3. CEM, $V_{m}$~ MWT, ${CL}_{bo}$ ~ BWT | 12833.8 | 12792.2 |

Figure S3.1. Mother volume of distribution $V_{m}$ vs mother’s weight

Figure S3.2. Baby water clearance ${CL}_{bo}$ vs baby’s weight
