# Supplementary material for: Development of a nonlinear hierarchical model to describe the disposition of deuterium in mother–infant pairs to assess exclusive breastfeeding practice
Source: J Pharmacokinet Pharmacodyn. 2018 Nov 14;46(1):1–13. doi: 10.1007/s10928-018-9613-x (PMC6394541; doi:10.1007/s10928-018-9613-x)
Supplement: Supplementary file 4 — Supplementary material 4 (DOCX 16 kb) [file 10928_2018_9613_MOESM4_ESM.docx]

**Supplement 4. Stan code**

data { # block declaration of the data

int<lower=1> N; # number of subjects

int<lower=1> Nobs; # number of observations

vector[Nobs] y; # observed concentration

vector[Nobs] time; # sampling time

vector[Nobs] dose; # dose

vector[Nobs] flag; # separate mother (0) and infant (1)

vector[Nobs] Wt_s; # infant weight in the beginning of study

vector[Nobs] Wt_e; # infant weight at the end of study

vector[N] MWT; # mother weight

vector[N] BWT; # infant average weight during the study

int<lower = 1> Tstart[N]; # time index number

int<lower = 1> Tstop[N]; # time index number

}

transformed data {

vector[Nobs] Wt; # infant weight increases with time

# linear increase with time assumed here

for (i in 1:N) {

for (j in Tstart[i]:Tstop[i]) {

Wt[j] = Wt_s[j] + (Wt_e[j]-Wt_s[j])*(time[j])/(time[Tstop[i]]);

}

}

}

parameters {

vector[4] log_theta_1; # priors of log_theta (i.e. Vm, Kmm, CLmb, CLbo in log domain)

vector<lower = 0>[4] omega_vec; # variance parameter

corr_matrix[4] rho; # correlation parameter

vector[4] phi[N]; # estimated parameters in log domain incorporating omega

vector<lower = 0>[2] sigma; # combined error model parameters

vector[2] theta; # estimated parameters related to the covariates

real a; # parameter to calculate infant total body water

real b; # parameter to calculate infant total body water

real waterinmilk; # free water proportion in breastmilk

real metabowaterinmilk; # metabolized water proportion in breastmilk

real absorptionproportion; # non-oral water intake proportion by absorption

}

transformed parameters {

real Cl_mb_Rs; # estimated CLmb to calculate Rs

real Cl_bo_Rs; # estimated CLbo to calculate Rs

real correctionfactor; # correction factor of total infant's water output rate

vector[N] M; # breastmilk mass in kg

vector[N] Rm_Rs; # metabolised water intake rate

vector[N] Rg_Rs; # water consumed by growth

vector[N] Rc_bo_Rs; # total baby water output after correction

vector[N] Ra_Rs; # absorption of atmospheric water

vector[N] Rs; # ingested water intake rate from sources other than breastmilk

vector[Nobs] Vb_b; # infant D2O volume of distribution

cov_matrix[4] Omega; # variance covariance matrix

Omega = quad_form_diag(rho, omega_vec); # incorporating variance and correlation parameters

for (j in 1:Nobs) {Vb_b[j] = 1.041*exp(a + b*log(Wt[j]));}

for (i in 1:N) {Rg_Rs[i]=(1/1.041)*(Vb_b[Tstop[i]]-Vb_b[Tstart[i]])/(time[Tstop[i]]-time[Tstart[i]]);}

correctionfactor=0.9906;

for (i in 1:N) {

Cl_mb_Rs = exp(phi[i,3]);

Cl_bo_Rs = exp(phi[i,4])*pow(BWT[i]/5,theta[2]);

M[i]=Cl_mb_Rs/waterinmilk;

Rm_Rs[i]=metabowaterinmilk*M[i];

Rc_bo_Rs[i]=Cl_bo_Rs/correctionfactor;

Ra_Rs[i]=absorptionproportion*(Rc_bo_Rs[i] + Rg_Rs[i]);

Rs[i]=1000*(Rc_bo_Rs[i]+Rg_Rs[i]-Cl_mb_Rs-Rm_Rs[i]-Ra_Rs[i]);

}

}

model { # block declaration of the model to be fitted

vector[Nobs] ipred; # predicted concentration

vector[4] log_theta; # estimated parameters

real Vm; # mother D2O volume of distribution

real kmm; # rate constant, describing D2O total elimination from the mother compartment

real Cl_mb; # water clearance rate from mother to infant

real Cl_bo; # water clearance rate from infant

real flag_g; # covariate defining mother (0) and infant (1)

real t; # time

real amt; # dose

log_theta_1 ~ normal(0, 1000); # priors

rho ~ lkj_corr(1); # priors

omega_vec ~ normal(0, 1000); # priors

a ~ normal(-0.427, 0.012); # priors

b ~ normal(0.963, 0.005); # priors

sigma ~ normal(0, 1000); # priors

theta ~ normal(0, 1000); # priors

waterinmilk ~ normal(0.889,0.0013); # priors

metabowaterinmilk ~ normal(0.07733, 0.00117); # priors

absorptionproportion ~ normal(0.063,0.017); # priors

for (i in 1:N) {

log_theta = log_theta_1;

phi[i]~ multi_normal(log_theta, Omega);

Vm = exp(phi[i,1])*pow(MWT[i]/70,theta[1]);

kmm = exp(phi[i,2]);

Cl_mb = exp(phi[i,3]);

Cl_bo = exp(phi[i,4])*pow(BWT[i]/5,theta[2]);

for (j in Tstart[i]:Tstop[i]) {

amt = dose[j];

t = time[j];

flag_g = flag[j];

ipred[j] = (1-flag_g)*(amt*1000/Vm)*exp(-kmm*t) + flag_g*(amt*1000/Vm)*(Cl_mb/(Vb_b[j]*(kmm-(Cl_bo/Vb_b[j]))))*(exp(-(Cl_bo/Vb_b[j])*t)-exp(-kmm*t));

y[j] ~ normal(ipred[j], pow((ipred[j]*ipred[j]*sigma[1]*sigma[1] + sigma[2]*sigma[2]), 0.5));

}

}

}

generated quantities { # block declaration to save model predictions

real Vm;

real kmm;

real Cl_mb;

real Cl_bo;

vector[Nobs] pred;

vector[Nobs] log_lik;

for (i in 1:N) {

for (j in Tstart[i]:Tstop[i]) {

Vm = exp(phi[i,1])*pow(MWT[i]/70,theta[1]);

kmm = exp(phi[i,2]);

Cl_mb = exp(phi[i,3]);

Cl_bo = exp(phi[i,4])*pow(BWT[i]/5,theta[2]);

pred[j] = (1-flag[j])*(dose[j]*1000/Vm)*exp(-kmm*time[j]) + flag[j]*(dose[j]*1000/Vm)*(Cl_mb/(Vb_b[j]*(kmm-(Cl_bo/Vb_b[j]))))*(exp(-(Cl_bo/Vb_b[j])*time[j])-exp(-kmm*time[j]));

log_lik[j] = normal_log(y[j], pred[j], pow((pred[j]*pred[j]*sigma[1]*sigma[1] + sigma[2]*sigma[2]), 0.5));

}

}

}
