# Supplementary material for: Development of a nonlinear hierarchical model to describe the disposition of deuterium in mother–infant pairs to assess exclusive breastfeeding practice
Source: J Pharmacokinet Pharmacodyn. 2018 Nov 14;46(1):1–13. doi: 10.1007/s10928-018-9613-x (PMC6394541; doi:10.1007/s10928-018-9613-x)
Supplement: Supplementary file 6 — Supplementary material 6 (DOCX 12 kb) [file 10928_2018_9613_MOESM6_ESM.docx]

**Supplement 6**

Table S6.1 Estimated population parameter values

| parameters | population mean | BSV % |
| --- | --- | --- |
| Vm (L) | 32.86 | 7.0 |
| Kmm (1/day) | 0.109 | 15.0 |
| CLmb (L/day) | 0.733 | 20.1 |
| CLbo (L/day) | 0.864 | 17.1 |
| Rs (g/day) | 50.7 | 57.1 |

| Vm: D2O volume of distribution in mother compartment |
| --- |
| Kmm: rate constant, describing D2O total elimination from mother compartment |
| CLmb: water clearance rate from mother to infant |
| CLbo: water clearance rate from infant |
| Rs: water intake from non-breastmilk sources |
| BSV: Between Subject Variability |
