# Supplementary material for: Development of a nonlinear hierarchical model to describe the disposition of deuterium in mother–infant pairs to assess exclusive breastfeeding practice
Source: J Pharmacokinet Pharmacodyn. 2018 Nov 14;46(1):1–13. doi: 10.1007/s10928-018-9613-x (PMC6394541; doi:10.1007/s10928-018-9613-x)
Supplement: Supplementary file 7 — Supplementary material 7 (DOCX 12 kb) [file 10928_2018_9613_MOESM7_ESM.docx]

**Supplement 7. Theoretical lower limit of** $\boldsymbol{R}_{\boldsymbol{s}}$

The purpose of this calculation is to identify the theoretical lower limit of $R_{s}$. This calculation is based on the assumptions that are inherent in the DTM method with a theoretical perfect sampling protocol (i.e. 7 samples collected crossing a 14-day study period) and perfect model (i.e. the best final model identified in this study as items 3 in Table S3.1). $R_{s}$is calculated as equation A1.3. After we fit the best final model with the calibration data set, we obtained the two primary parameters as below:

$${log(CL}_{bo})\sim N(-0.16, 0.17)$$

$${log(CL}_{mb})\sim N(-0.33, 0.20)$$

Note:

1) $N(mean, SD)$; mean as population mean, SD as Between Subject Variability;

2) correlation between ${CL}_{bo}$ and ${CL}_{mb}$ is 0.99

The values (as normal distributions) of the other four parameters ($R_{c\left( bo \right)},R_{g}, R_{m},R_{a}$) in equation A1.3 are introduced in detail in the subsection “Statistical models”.

From each parameter’s distribution, ten thousand random samples were taken and the statistical inference of $R_{s}$(e.g. mean, 5% and 95% percentiles) can be calculated. The 95% upper bound on $R_{s}$for perfect EBF mothers is approx. 56 g/day.
